# Oncologic Imaging of the Lymphatic System: Current Perspective with Multi-Modality Imaging and New Horizon

**DOI:** 10.3390/cancers13184554

**Published:** 2021-09-10

**Authors:** Mohamed Elshikh, Ahmed W Moawad, Usama Salem, Sergio P Klimkowski, Talaat Hassan, Brinda Rao Korivi, Corey T Jensen, Sanaz Javadi, Khaled M Elsayes

**Affiliations:** 1 Department of Diagnostic and Interventional Radiology, The University of Texas Medical Branch, Galveston, TX 77555, USA; 2 Department of Diagnostic and Interventional Radiology, Mercy Catholic Medical Center, Darby, PA 19023, USA; ahmedw.moawad@mercyhealth.org; 3 Department of Abdominal Imaging, The University of Texas MD Anderson Cancer Center, Houston, TX 77030, USA; USalem@mdanderson.org (U.S.); SPKlimkowski@mdanderson.org (S.P.K.); BRRao@mdanderson.org (B.R.K.); CJensen@mdanderson.org (C.T.J.); Sanaz.Javadi@mdanderson.org (S.J.); 4 Department of Plastic Surgery, Kasr Alainy School of Medicine, Cairo University, Cairo 11562, Egypt; Talaat.hassan@kasralainy.edu.eg

**Keywords:** lymphatic system imaging, lymphatic system physiology, lymphatic system anatomy, lymphatic metastasis, lymphangiography, lymphoscintigraphy, magnetic resonance lymphangiography, conventional lymphography

## Abstract

**Simple Summary:**

The lymphatic system is an essential component of the human circulatory system that plays a critical role in antigen presentation, mounting immune reactions, gastrointestinal tract lipid absorption, and maintenance of interstitial homeostasis. This complex network of specially adapted vessels and lymphoid organs also represents major pathway for cancer spread. Knowledge of lymphatic anatomy, physiology, and expected imaging appearances is crucial in understanding the pattern of cancer spread, with great implications for treatment and management. In this review article, we discuss lymphatic anatomy, physiology, imaging techniques, and radiographic appearances of cancer spread with relevant illustrative cases.

**Abstract:**

The lymphatic system is an anatomically complex vascular network that is responsible for interstitial fluid homeostasis, transport of large interstitial particles and cells, immunity, and lipid absorption in the gastrointestinal tract. This network of specially adapted vessels and lymphoid tissue provides a major pathway for metastatic spread. Many malignancies produce vascular endothelial factors that induce tumoral and peritumoral lymphangiogenesis, increasing the likelihood for lymphatic spread. Radiologic evaluation for disease staging is the cornerstone of oncologic patient treatment and management. Multiple imaging modalities are available to access both local and distant metastasis. In this manuscript, we review the anatomy, physiology, and imaging of the lymphatic system.

## 1. Introduction

The lymphatic system is an anatomically complex vascular system that is involved in mounting immune reactions, maintaining interstitial fluid homeostasis, and transporting large interstitial particles and cells, as well as in gastrointestinal tract lipid absorption [1,2,3]. The lymphatic system is composed of lymphatic organs that are interconnected with lymphatic vessels. Lymphatic organs include organs such as lymph nodes, spleen, thymus, and bone marrow [1]. This network of specially adapted vessels and lymphoid tissue provides a major pathway for cancer spread. Many tumors express vascular endothelial factors that induce neoangiogenesis and lymphangiogenesis. These processes induce increased growth of blood and lymphatic vessels to the tumor and surrounding regions, providing additional nutrients for tumor growth and more pathways for spread. Increased lymphangiogenesis is associated with higher probability of metastasis through the lymphatic system, including lymph nodes [4,5,6,7]. The most commonly used cancer staging system is the TNM system, where N represents lymph node status. Accurate determination of the N-stage is critical for patient treatment and management. Multiple imaging modalities are utilized to accurately stage disease including ultrasound (US), computed tomography (CT), magnetic resonance imaging (MRI), positron emission tomography (PET), positron emission tomography–computed tomography (PET-CT), conventional lymphangiography (CL), magnetic resonance lymphangiography (MRL), and sentinel lymph node (SNL) imaging [8,9,10]. In this review, we briefly illustrate the structure, anatomy, and the most commonly used imaging modalities for evaluation of the lymphatic system.

## 2. Microscopic Anatomy and Physiology of the Lymphatic System

**Figure 1:**
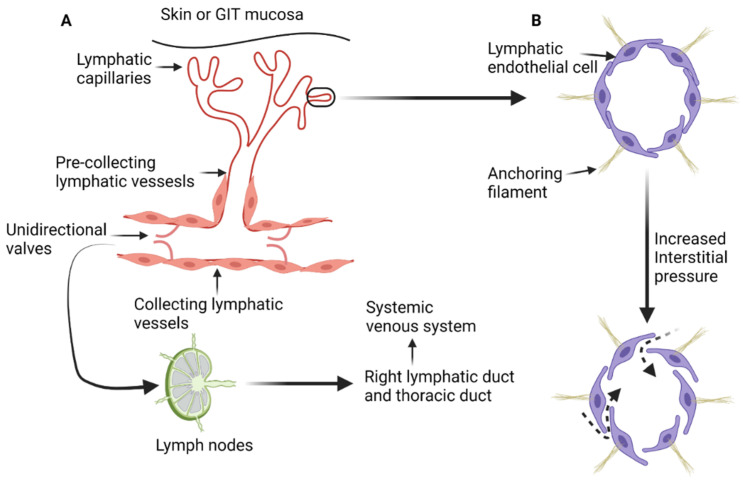
Diagrammatic illustration of the anatomy and physiology of the lymphatic system. (**A**) The blind end lymphatic capillaries that drain in the pre-collecting lymphatic vessels. Lymph is then transferred to the collecting lymphatic vessels and get filtered by the lymph nodes. All lymph from the human body ends in the systemic venous system through either the thoracic duct or right lymphatic duct. (**B**) The effect on increased interstitial pressure on the loose junctions between lymphatic endothelial cells, leading to increased transmission of the interstitial fluid and proteins to the lymphatic capillaries and pre-collecting lymphatic vessels (dashed arrows). “Created by BioRender.com. accessed on 10 August 2021”.

The lymphatic system is composed of a network of lymphatic vessels that connect lymph nodes and lymphoid organs. Lymphoid organs can be classified as primary or secondary. Primary lymphoid organs such as the thymus and bone marrow are involved in production of lymphocytes. The secondary lymphoid organs such as the spleen, lymph nodes, and tonsils control the maturation of lymphocytes and regulate immune response to pathogens and/or allergens [1,2]. In addition, the lymphatic system maintains fluid homeostasis in the interstitial compartment and participates in the absorptive function of the gastrointestinal tract [1,3]. The lymphatic system starts with blind-ended lymph capillaries (Figure 1A) in the skin and mucosa of the gastrointestinal tract. These capillaries are lined by a single layer of endothelial cells that are loosely connected to each other and deficient in both the basement membrane and pericytes. The loose connections between lymphatic endothelial cells increase the permeability of the lymphatic capillaries and facilitate exchange with the interstitial compartment [1,2,4]. Lymphatic endothelial cells are connected to the extracellular interstitial tissue with anchoring filaments [4,11]. When the interstitial tissue pressure increases, the anchoring filaments stretch and the junctions between lymphatic endothelial cells widen, resulting in increased passive transport (Figure 1B) of interstitial fluid, particles, lipids, and proteins [1,2,4].

Lymphatic capillaries drain into pre-collecting lymphatic vessels, which are also lined by a single layer of lymphatic endothelial cells and occasionally are surrounded by a single layer of smooth muscle [1,12]. Pre-collecting lymphatic vessels transport lymph to the collecting lymphatics that comprise of intima, media, and adventitia layers (layered anatomy found in blood vessels). The endothelial cells in collecting lymphatics are tightly adherent together with zipperlike junctions and are surrounded by a basement membrane to prevent lymph leakage. Collecting lymphatics have unidirectional valves that permit only forward flow of lymph toward the heart [12]. Eventually, lymph drains into the systemic venous system through either the right lymphatic duct or the thoracic duct. Transit of lymph throughout lymphatic system is characterized by passage through lymph nodes and lymphoid tissue where complex processes take place such as antigen presentation and activation/modulation of immune system. This process also presents a pathway for deposition of tumor cells in the lymphoid tissue along the way, resulting in metastatic spread. Lymph nodes receive multiple afferent lymph vessels through the cortex and drain through efferent lymphatic vessels [2].

### 2.1. Lymphatic Anatomy of the Abdomen, Pelvis and Lower Extremities

#### 2.1.1. Inguinal and Popliteal Lymph Nodes and Lower Extremity Lymphatic Drainage Pathways

**Figure 2:**
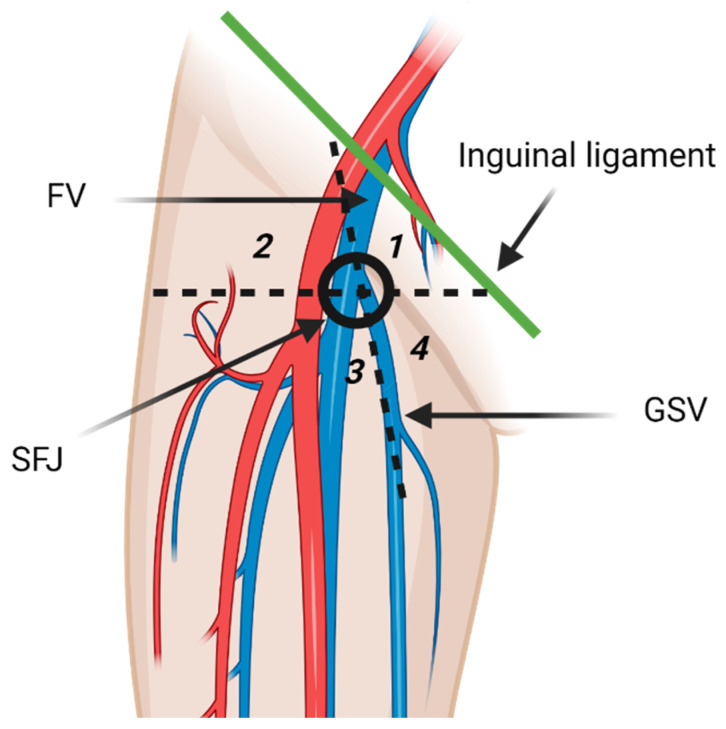
Diagrammatic illustration of the superficial inguinal lymph nodes anatomy. The superficial inguinal lymph nodes are inferior to the inguinal ligament and divided in five groups by the greater saphenous vein (GSV) and a horizontal line through the saphenofemoral junction (SFJ). The five groups are superior medial (**1**), superior lateral (**2**), inferior lateral (**3**), inferior medial (**4**), and central group that overlies the SFJ. FV: femoral vein. The inferolateral group receives most of the lymphatic drainage of the lower extremity. “Created by BioRender.com. accessed on 10 August 2021”.

The inguinal lymph nodes are classified as superficial and deep nodes on the basis of the relationship to the fascia lata [3]. The superficial inguinal lymph nodes are further classified into five subgroups by the greater saphenous vein (GSV) and a horizontal line running through the saphenofemoral junction (Figure 2). The five subgroups include superomedial, superolateral, inferomedial, inferolateral, and central groups [3,13]. The superomedial group drains the medial infraumbilical abdominal wall, medial gluteal region, external genitalia, lower anal canal, and the perianal region. The superolateral group receives lymph from lateral infraumbilical region, lower back, and lateral gluteal region [3]. Lymphatic drainage pathways of lower extremities are comprised of two distinct routes that may communicate the posterolateral and the anteromedial lymphatic vessels. The posterolateral pathway follows the small saphenous vein and drains in the popliteal lymph nodes. Efferent lymphatic vessels from the popliteal lymph nodes either join the deep lower extremity lymphatic system and terminate in the deep inguinal lymph nodes, or join the anteromedial lymphatic vessels [14]. Anteromedial lymphatic pathway continues with the great saphenous vein and usually drains in the inferolateral group [13,14]. Deep inguinal lymph nodes drain the lower extremity deep lymphatic vessels that accompany the femoral vessels [3]. Efferent vessels from the inguinal lymph nodes transport lymph to the external iliac lymph nodes [3]. Some lymphatic vessels from the lower extremity bypass the superficial and deep inguinal lymph nodes and drain directly in the external iliac lymph nodes. Meticulous examination of the external iliac lymph nodes should be routinely performed on patients with lower extremity malignancies even if the inguinal lymph nodes are normal [14].

#### 2.1.2. Iliac Lymph Nodes Anatomy

**Figure 3:**
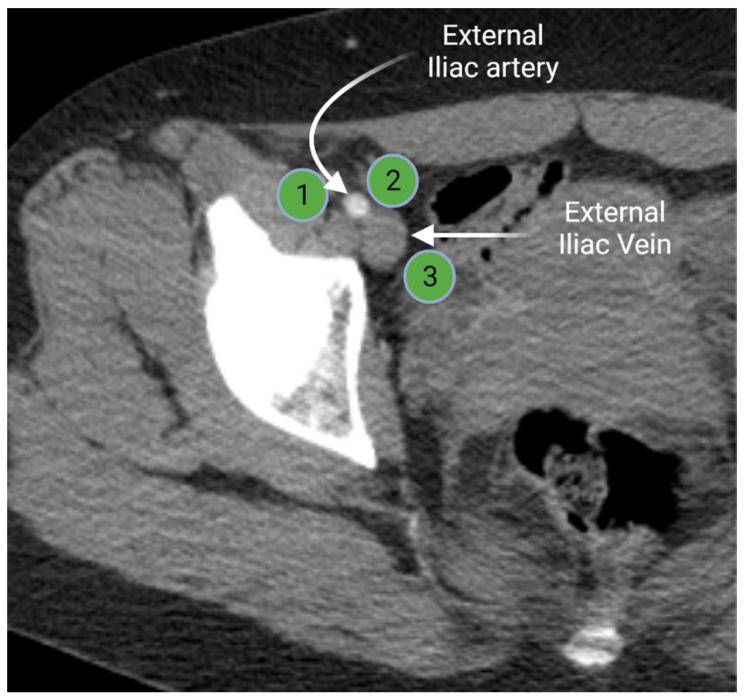
Axial contrast-enhanced computed tomography of the right hemipelvis demonstrates the right external iliac artery and veins with associated lateral (**1**), middle (**2**), and medial (**3**) external iliac lymph nodes. “Created by BioRander.com. accessed on 10 August 2021”.

Iliac lymph node chain includes the external, internal, and common iliac lymph nodes. The external iliac lymph nodes are in close proximity to the external iliac vessels. The location of these nodes extends from the bifurcation of the common iliac vessels to the inguinal ligament [3,15]. The external iliac lymph nodes are divided in three subgroups (lateral, middle, and medial) according to their position relative to the external iliac vessels (Figure 3). The medial group is located medial or posteromedial to the external iliac vessels. The medial group is sometimes referred to as the obturator lymph nodes. The middle group lies between the external iliac artery and vein. The lateral group is lateral to the external iliac artery [15,16]. The external iliac lymph nodes provide the drainage route for the inguinal lymph nodes [3,16].

**Figure 4:**
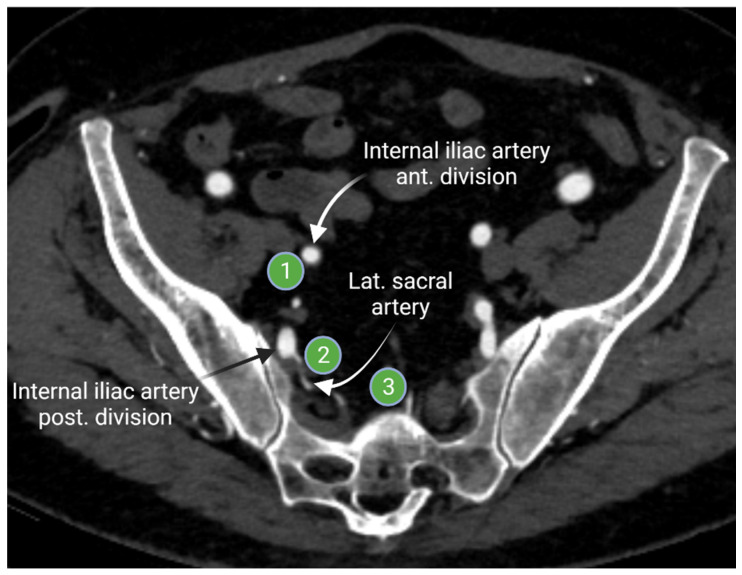
Axial contrast-enhanced computed tomography of the pelvis shows the anterior internal iliac lymph node group (**1**) that follows the course of the internal iliac artery anterior division, the lateral sacral lymph node group (**2**) that runs along the lateral sacral artery, and the presacral lymph nodes (**3**) that lies in the midline presacral region. “Created by BioRander.com. accessed on 10 August 2021”.

The internal iliac lymph nodes drain the pelvic organs and follow the branches of the internal iliac artery. These lymph nodes are sometimes referred to as the hypogastric lymph nodes, which are located posteriorly in the pelvis. The internal iliac lymph chain includes multiple lymph node groups. Most identified internal iliac lymph node groups include the anterior iliac, lateral sacral, and presacral lymph nodes. The anterior internal iliac lymph nodes run along the anterior division of the internal iliac artery (Figure 4). The presacral lymph nodes are in the midline anterior to the sacrum. The lateral sacral lymph nodes follow the lateral sacral artery (Figure 4) [15,16].

**Figure 5:**
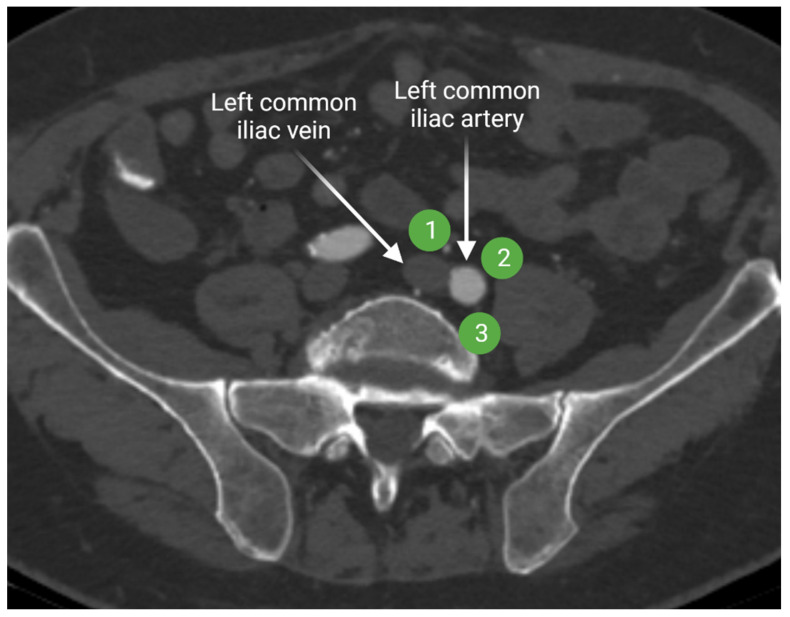
Axial contrast-enhanced CT of the upper pelvis at the level of the common iliac vessels shows the common iliac artery and vein. The lateral common iliac lymph node subgroup (**1**) is located lateral to the common iliac artery, while the medial subgroup (**2**) is medial to the common iliac artery. The middle subgroup (**3**) is in the lumbosacral fossa. “Created by BioRender.com. accessed on 10 August 2021”.

Lymph from the internal and external iliac lymphatic chains drains more centrally to the common iliac lymph nodes. The common iliac lymph nodes follow the course of the common iliac vessels and extend from the aortic bifurcation to the common iliac bifurcation [15,16]. Common iliac lymph nodes are divided in three subgroups: lateral, medial, and middle lymphatic chains (Figure 5). The lateral and medial subgroups are located on the lateral and medial sides of the common iliac artery, respectively. The middle subgroup is in the lumbosacral fossa. Lumbosacral fossa is anterior to the lower lumbar and proximal sacral vertebrae and bordered anterolaterally by the iliopsoas muscle.

#### 2.1.3. Peri-Aortic Lymph Node Anatomy

**Figure 6:**
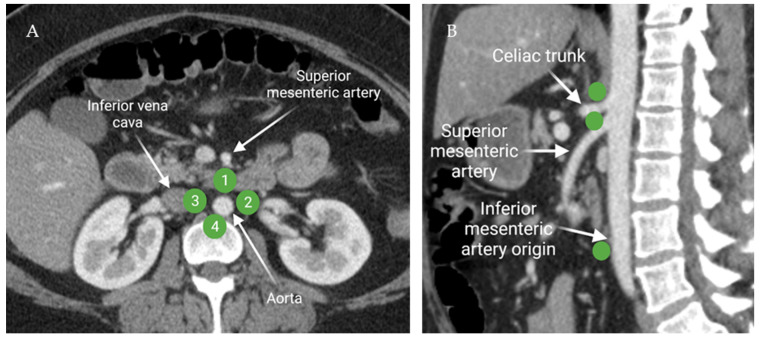
Axial contrast-enhanced CT of the abdomen (**A**) demonstrates the distribution of the peri-aortic lymph nodes: **1**; pre-aortic, **2**; left lateral aortic, **3**; right lateral aortic, and **4**; post-aortic. Sagittal contrast-enhanced CT of the abdomen (**B**) shows the distribution of the pre-aortic lymph nodes along the origins of the visceral branches of the aorta (celiac, superior mesenteric, and the inferior mesenteric arteries). “Created by BioRender.com. accessed on 10 August 2021”.

The common iliac lymph nodes transport lymphatic drainage from the lower extremities and pelvis to the retroperitoneal lymph nodes. Functionally, retroperitoneal lymph nodes are categorized in four subgroups: pre-aortic, right and left lateral aortic, and post-aortic lymph nodes (Figure 6). The pre-aortic lymph nodes are anterior to the abdominal aorta around the visceral branches (celiac, superior mesenteric, and inferior mesenteric arteries). They receive afferent lymphatics from the bowel. The right and left lateral aortic lymph nodes receive efferent lymphatics from the ipsilateral iliac lymph nodes, kidney, adrenal gland, and the gonads. The post-aortic subgroup shares the drainage with the lateral subgroups. Afferent lymphatics from the peri-aortic lymph nodes transport lymph to the cisterna chyli [17].

#### 2.1.4. Anatomy of the Visceral/Digestive Lymph Nodes

**Figure 7:**
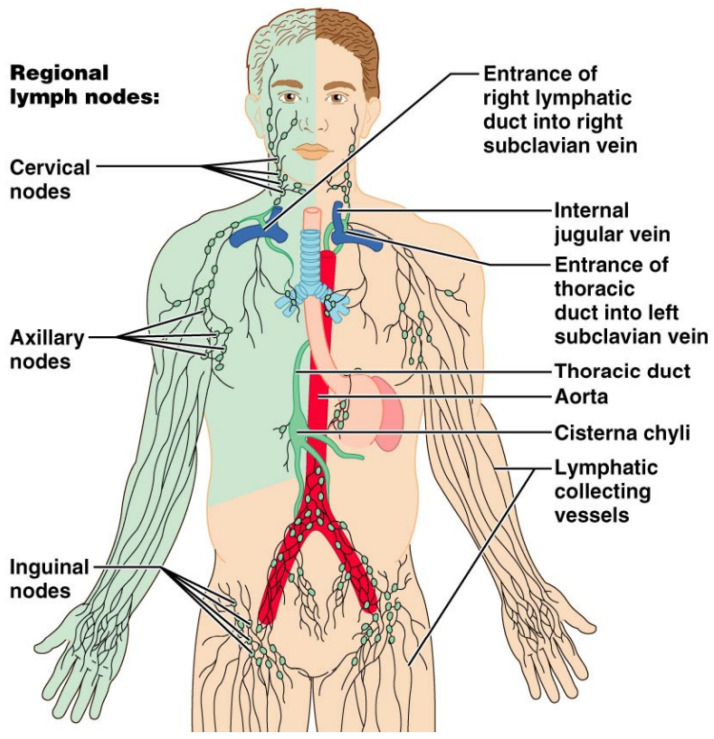
Diagrammatic illustration of the lymphatic drainage of the whole human body. The right upper extremity, right hemiface, and right hemithorax are drained by the right lymphatic duct (green-colored). The thoracic duct drains the rest of the human body.

The digestive tract lymph is drained through the pre-aortic lymph nodes that include three distinct lymph nodes stations running along the origin of the visceral branches of the aorta. These lymph stations are the celiac, superior mesenteric, and inferior mesenteric lymph nodes. The celiac lymph nodes receive afferent lymphatics from the stomach, duodenum, pancreas, spleen, and most of the hepatobiliary system [3,17]. The superior mesenteric lymph nodes run along the superior mesenteric artery branches. These lymph nodes drain the distal duodenum, ileum, jejunum, ascending colon, and proximal transverse colon. The inferior mesenteric lymph nodes drain the rest of the colon and the upper rectum [17]. These visceral lymph nodes drain into the cisterna chyli, which eventually drains into the thoracic duct that provides drainage of the left hemithorax, abdomen, and lower extremities. Thoracic duct enters the systemic venous system at the left subclavian and internal jugular vein junction. On the other hand, the right lymphatic duct drains lymph from the right hemithorax, right upper extremity, and right hemiface (Figure 7) [3,18].

## 3. Lymphatic Spread of Cancer

The lymphatic system provides a major pathway for tumor spread. There is proven correlation between tumoral lymphangiogenesis, invasion of the lymphatic system, and distant/hematogenous metastasis [4,5]. Vascular endothelial growth factors (VEGF)-C and -D have been associated with high metastatic potential to the lymphatic system. VEGF-C and -D induce lymphangiogenesis through activation of vascular endothelial growth factor receptor-3 (VEGFR-3), which is mainly produced by the lymphatic endothelial cells. Tumors overexpressing VEGF-A, neutropilin-2, VEGF-C, and VEGF-D are associated with increased potential for lymphatic spread [4,5,6,7,19].

## 4. Radiologic Evaluation of the Lymphatic System in Cancer Patients

Radiologic evaluation of the lymphatic system is challenging due to the complex anatomy and physiology. Lymphatic system is composed of lymphoid organs interconnected together with the lymphatic vessels. Broadly speaking, lymphoid organs such as lymph nodes and spleen are characterized by anatomic and physiologic imaging such as computed tomography (CT), magnetic resonance (MR), ultrasound (US), or positron emission tomography (PET) [8]. On the other hand, lymphatic vessels are evaluated by magnetic resonance lymphangiography or conventional lymphangiography [8,9].

Cancer treatment relies greatly on the TNM staging, where the N represents the nodal stage of the tumor. Cross-sectional anatomic imaging has been the cornerstone modality to determine the N stage of most cancers. Anatomic imaging depends heavily on lymph nodes size criteria and morphology to determine the probability of lymphatic metastasis. However, this approach can be problematic because small and anatomically normal lymph nodes can harbor micro-metastases beyond the capability of anatomical imaging. Functional imaging such as positron emission tomography (PET), ultrasmall superparamagnetic particles of iron oxide (USPIO) MRI, and contrast-enhanced US have been used for cancer staging in these situations [9,10].

### 4.1. Cross-Sectional Evaluation of the Lymphatic System

#### 4.1.1. Ultrasound Evaluation of the Lymphatic System

**Figure 8:**
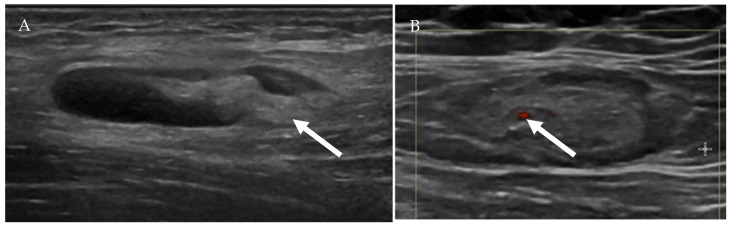
Ultrasound of a benign-appearing cervical lymph node (**A**) shows an oval hypoechoic lymph node with echogenic fatty hilum (arrow). Color doppler ultrasound of another benign-appearing lymph node (**B**) demonstrates a fatty hilum (normal) with mild hilar vascularity (arrow).

**Figure 9:**
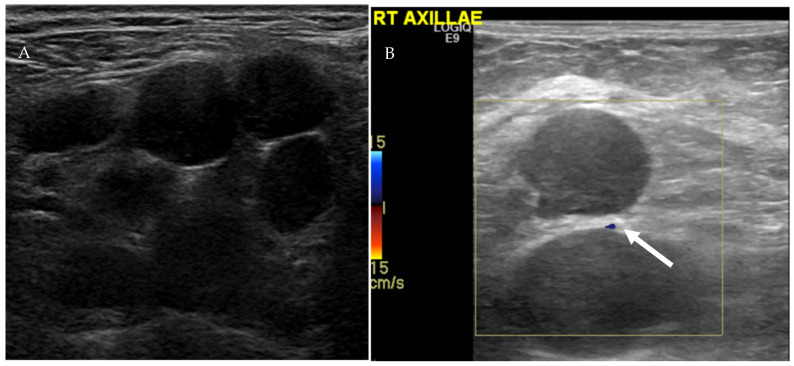
Ultrasound of the right axilla (**A**) demonstrates multiple, round, and hypoechoic metastatic lymph nodes with S/L axis ratio of approximately 1 and lack of the echogenic fatty hilum. Color Doppler ultrasound of the axillary lymph (**B**) nodes shows peripheral vascularity (arrow).

US is a noninvasive imaging modality that can be performed at bedside to evaluate nodal disease without use of ionizing radiation, with the possibility of biopsy and tissue diagnosis at the same time. Main disadvantages of US include operator dependency and poor penetration of deeper tissues [8]. There is no single US feature that can accurately discriminate between benign and malignant lymph nodes. Furthermore, there is a significant overlap in the imaging appearance between reactive and malignant lymph nodes. Nuanced approach using a combination of several US grayscale and Doppler imaging features has been suggested to differentiate between benign and malignant lymph nodes. Morphologic features such as lymph node size, shape, presence of hilar fat, echogenicity, vascular pattern, and flow resistance have been helpful in characterizing lymph nodes (Table 1) [20,21]. Ratio of short to long (S/L) lymph node axis can also be used to determine nodal benignity. S/L less than 0.5 is typically associated with benignity [22]. Benign lymph nodes are usually small (<1 cm in short axis), oval with smooth or indistinct margins, and contain echogenic fatty hila (Figure 8) [20,23]. On the other hand, malignant lymph nodes typically appear hypoechoic with effacement or infiltration of fatty hila, usually measure more than 1 cm in short axis with S/L axis ratio ≥ 0.5, and have a rounded shape (Figure 9) [20].

**Figure 10:**
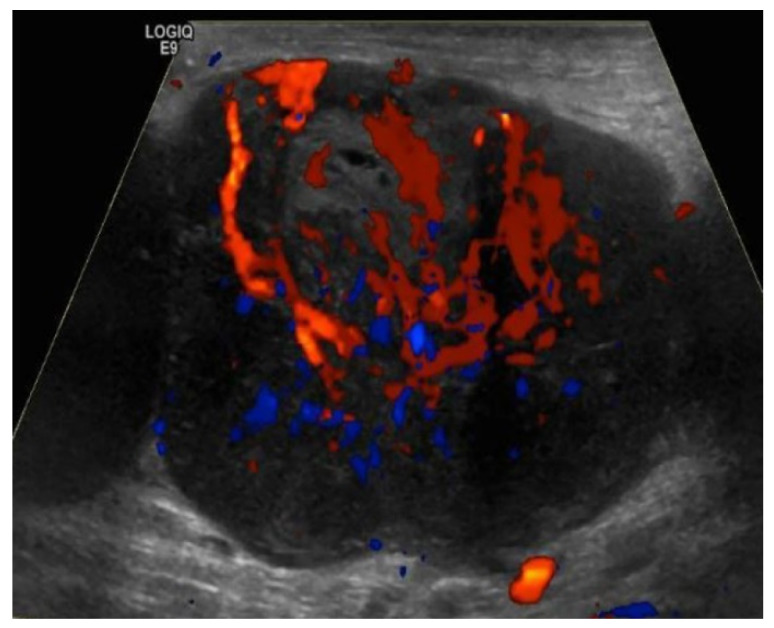
Doppler ultrasound of a right inguinal metastatic lymph node in a 67-year-old male with history of right ankle melanoma shows an enlarged hypoechoic lymph node with mixed (hilar and peripheral) vascularity.

Vascular pattern seen on Doppler can be used in conjunction with grayscale features to assess benignity. Four vascular patterns of lymph node blood supply have been described: hilar, peripheral, mixed (peripheral and hilar), and avascular [24]. Normal lymph nodes are either avascular or have hilar blood flow (Figure 8). Malignant or reactive lymph nodes vascularity is usually peripheral or mixed (Figure 9 and Figure 10) [21,24,25]. Vascular resistance and resistive indices may offer clues in discerning malignant from reactive lymph nodes. Reactive lymph nodes typically demonstrate low vascular resistance secondary to vasodilation. On the other hand, malignant lymph nodes are more likely to demonstrative high resistive indices due to malignant infiltration of the lymph nodes that increases intranodal pressure and compresses blood vessels [24].

Contrast-enhanced US (CEUS) is an US-based imaging modality that utilizes microbubbles as the contrast agent. CEUS increases the specificity, sensitivity, and accuracy of US in differentiating between benign and malignant lymph nodes to 93%, 92%, and 92.8%, respectively [26]. Neoplastic invasion of lymph nodes changes lymph node architecture due to proliferation of malignant cells. These changes include increase lymph node vascularity due to peri- and intra-nodal angiogenesis. When tumor growth exceeds the vascular supply, focal or diffuse necrosis occurs. These changes manifest as mixed or peripheral (centripetal) vascularity, lack of hilar enhancement, and focal or diffuse hypoenhancement [21,26,27,28,29,30,31,32]. In contrast, reactive lymph nodes usually demonstrate increased hilar enhancement (centrifugal) and lack of necrosis/hypoenhancement [21,26,28,30,31]. Despite the characteristic patterns of benign and malignant lymph nodes on CEUS, caution should be practiced when interpreting these findings. Lymphoma for example can mimic reactive lymph node enhancement characteristics. On the other hand, tuberculosis may resemble malignant lymph nodes on CEUS [27,30,33,34]. 

#### 4.1.2. Computed Tomography (CT) and Conventional Magnetic Resonance Imaging (MRI) of the Lymphatic System

Conventional CT and MRI are the most common imaging modalities used to determine the N-stage of most cancers. CT and MRI provide high anatomical detail and outperform US in evaluation of deeper lymph nodes. Lymph node morphology and size are the main determinant of malignant potential on CT and MRI [35]. There is no consensus on a cutoff size for metastatic lymph nodes. Classically, lymph nodes more than 10 mm are concerning. However, this rule varies by lymph node station and sometimes malignancy subtype. Normal inguinal lymph nodes can measure up to 15 mm in short axis [36]. However, retroperitoneal, and iliac chain lymph nodes are concerning when they exceed 6–8 mm [15,37,38,39,40,41]. In addition to lymph node size, morphology is a crucial factor to consider when evaluating for malignant involvement. Combining both size and morphology increases accuracy for malignant lymph node detection. Normal lymph nodes are oval, oblong, or kidney-shaped with fatty hila. Round morphology, loss of fatty hilum, and necrosis are worrisome features concerning for metastasis [15].

#### 4.1.3. Positron Emission Tomography (PET) and Positron Emission Tomography-Computed Tomography (PET-CT)

PET is a physiologic imaging modality that relies on injecting patients with an intravenous radiopharmaceutical agent that is taken-up and concentrated by the target tissue. Radiopharmaceutical agent is a combination of a radioactive isotope and a pharmaceutical agent. The isotope is the radioactive component of the radiopharmaceutical and responsible for releasing the energy through an annihilation reaction between the positrons and electrons in the target tissue releasing a pair of 511 KeV photons. The released photon pair is detected by PET detectors implanted in the PET gantry. Multiple isotopes are used for PET imaging including ^11^C, ^13^N, ^15^O, and ^18^F. The pharmaceutical component of the radiopharmaceutical agent determines tissue specificity. The most commonly used radiopharmaceutical agent in oncological imaging is 18-fluoro-deoxy-glucose (18F-FDG). PET is an excellent imaging modality that provides excellent staging information. However, relatively low spatial resolution has been the main disadvantage of PET imaging. This limitation has been partially overcome with the advent of hybrid physiologic and anatomic techniques such as positron emission tomography–computed tomography (PET-CT) and positron emission tomography–magnetic resonance (PET-MR). These allow for improved spatial resolution with improved performance in oncological imaging [42]. 

**Figure 11:**
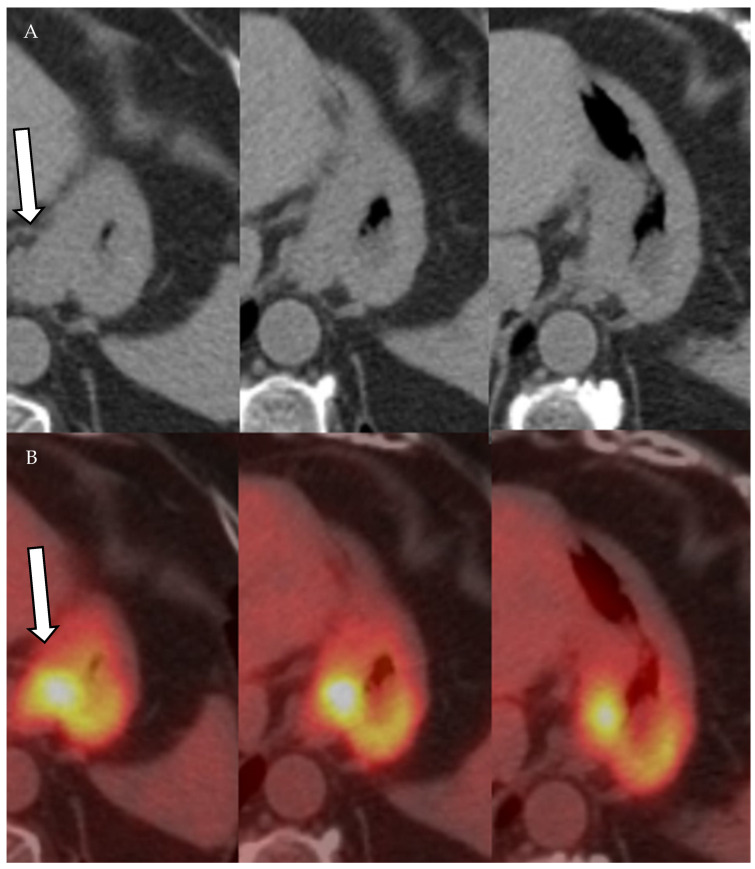
A 61-year-old female patient with history of gastric cancer. PET/CT scan demonstrates diffuse thickening of the gastro-esophageal region as well as the gastric cardia (arrow) (**A**) consistent with gastric carcinoma, which is FDG-avid (arrow) with maximum SUV = 11.6 (**B**). There is no focal abnormal metabolic activity to suggest distant metastatic spread or regional lymphadenopathy.

**Figure 12:**
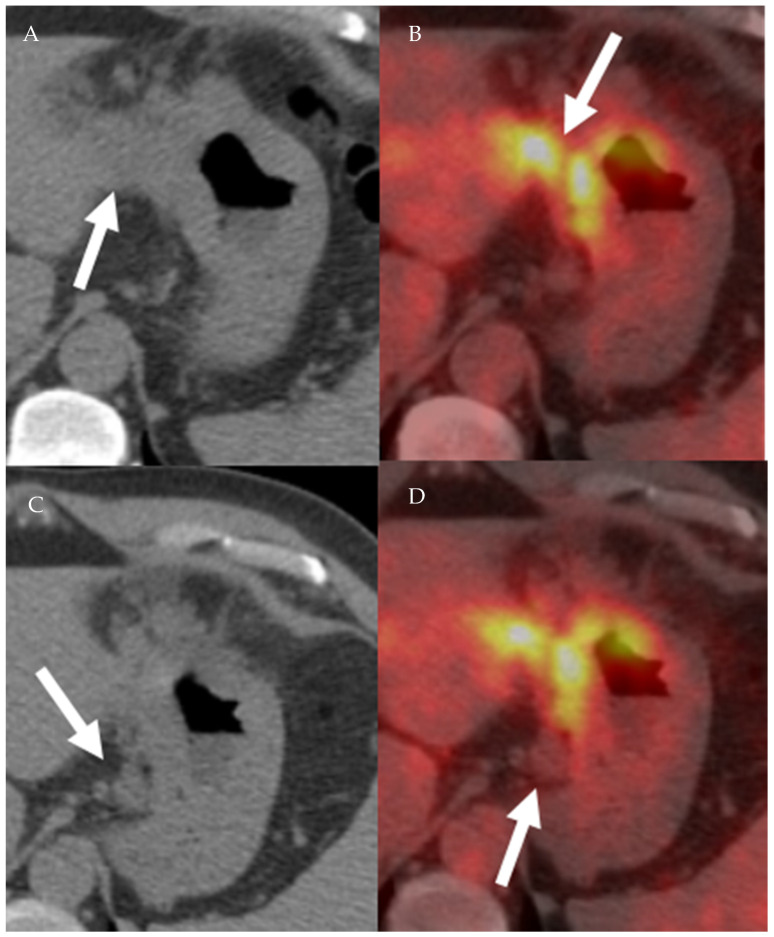
A 77-year-old male patient with gastric cancer. PET/CT scan demonstrates marked thickening of the gastric antrum with increased uptake (**A**,**B**) consistent with gastric carcinoma, maximum SUV = 8.7 (**B**). Enlarging metastatic adenopathy in the gastrohepatic ligament is also noted (**C**,**D**), not hypermetabolic on PET/CT.

**Figure 13:**
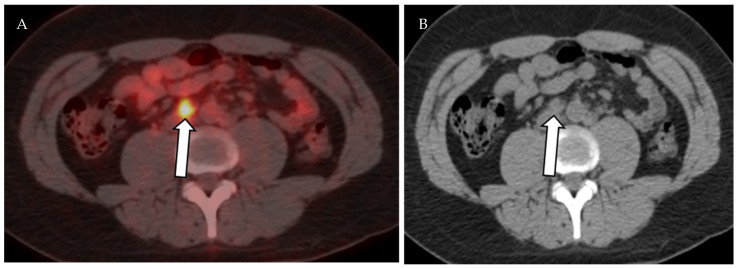
Axial fused PET-CT (**A**) and non-contrast CT of the abdomen shows mildly enlarged right paraaortic lymph node with increased FDG avidity on PET-CT (SUVmax 5.7), (**B**) biopsy proven Hodgkin lymphoma.

**Figure 14:**
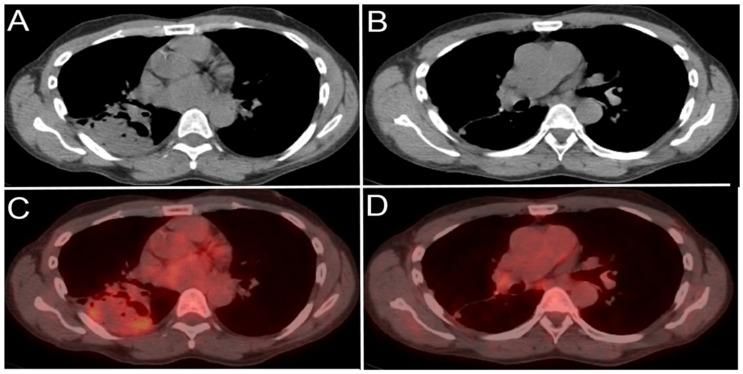
A 65-year-old male patient with right lower lobe lung cancer. The pulmonary mass seen in (**A**) shows avid FDG uptake (SUV = 4.6) in (**C**). Right hilar and subcarinal lymph nodes show mild FDG uptake (SUV = 2.6), which was found later to be inflammatory due to chemotherapy, resolving on (**B**,**D**). This is an example of false positive uptake in nonmalignant tissue.

FDG uptake in neoplastic cells depends on the expression of glucose transporters. Most cancer cells are hypermetabolic and overexpress glucose transporters. Hence, most cancer cells will demonstrate increased uptake of 18F-FDG (Figure 11, Figure 12 and Figure 13). After cellular uptake, 18F-FDG undergoes phosphorylation by hexokinase and becomes trapped inside the cell. In addition to hypermetabolic cancer cells, inflammatory cells have increased 18F-FDG uptake, resulting in decreased 18F-FDG PET-CT specificity (Figure 14) [43]. Moreover, PET and PET-CT performance in detecting primary and metastatic lesions varies according the FDG avidity of tumor cells [43]. In cervical cancer, PET and PET-CT have higher sensitivity and specificity than CT and MRI in diagnosing malignant lymph nodes [44,45]. In breast cancer, PET-CT is more sensitive and specific when compared to US in detecting metastatic axillary lymph nodes. Additionally, PET-CT changes management in approximately 13% of breast cancer patients [46,47].

### 4.2. Vascular Imaging of the Lymphatic System

#### 4.2.1. Conventional Lymphangiography (CL)

**Figure 15:**
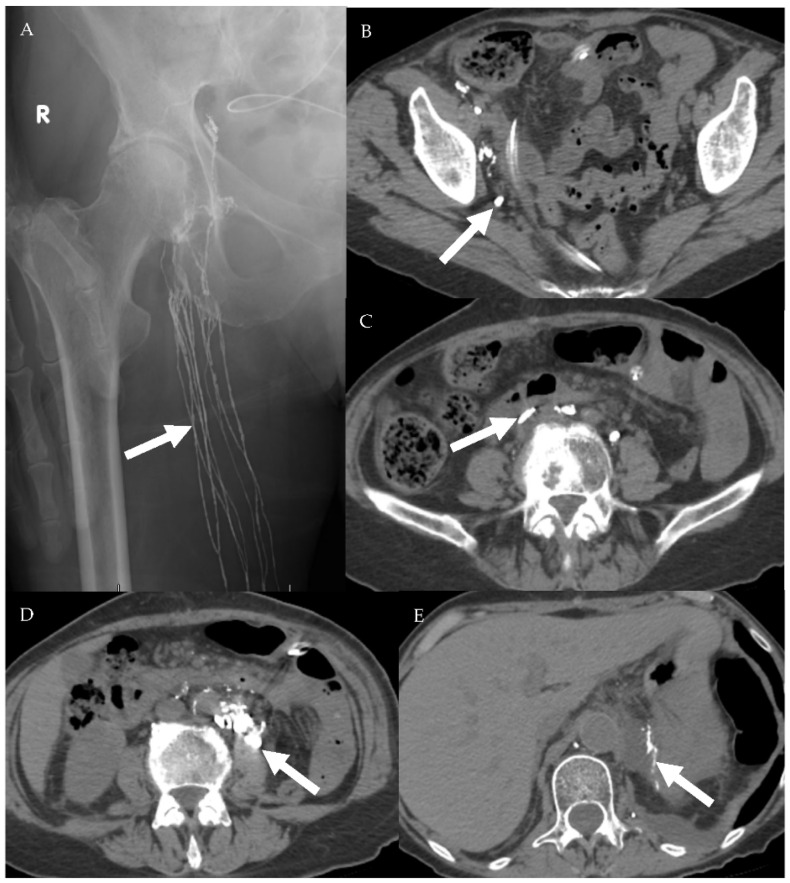
A 75-year-old female patient with abdominal leiomyosarcoma and chylous ascites. (**A**) Conventional lymphangiography was performed through the right pedal approach. The right inguinal lymphatic vessels are opacified (arrow) with subsequent opacification of the right pelvic lymphatic network (**B**,**C**). Noncontrast CT performed after lymphangiography shows ascending opacification of the lymphatic vessels and cisterna chyle (**D**). Estimated site of leakage appears from small lymphatic vessels in the left peri-aortic region at level of L3 (**E**).

**Figure 16:**
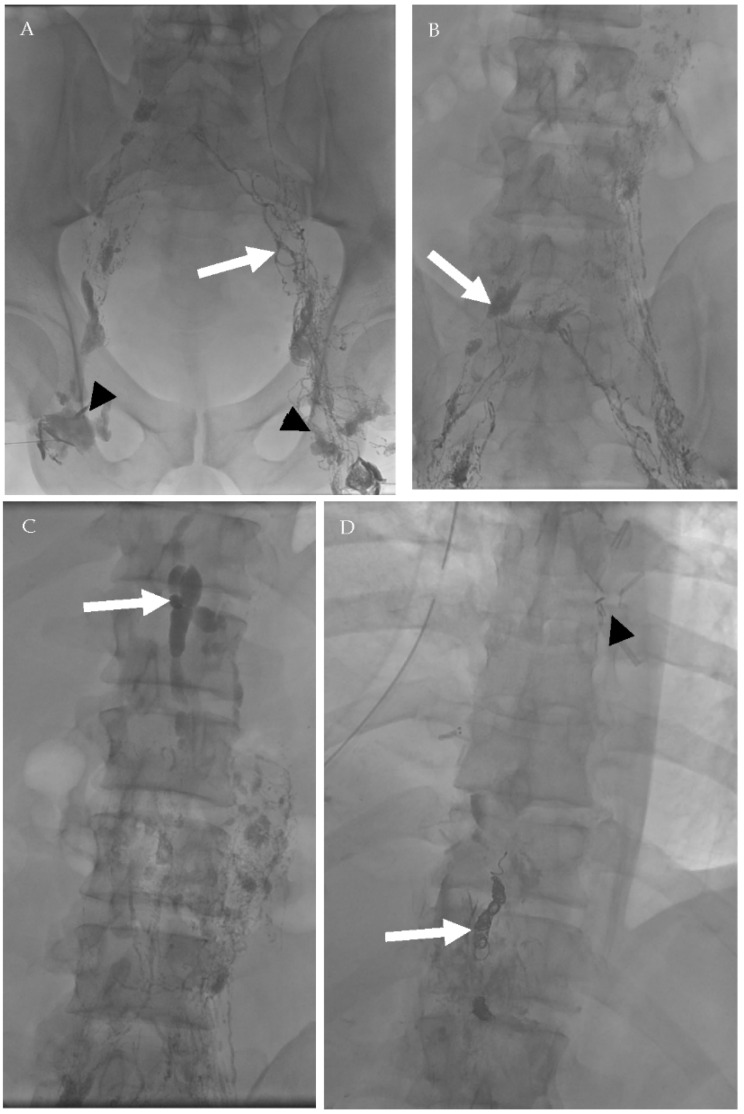
A 39-year-old male patient who underwent surgical resection of esophageal leiomyoma complicated by persistent chylous effusion. Successful percutaneous fluoroscopic-guided thoracic duct embolization using embolization coils. (**A**) Bilateral conventional lymphangiography was done through inguinal lymph nodes (arrowheads) with lymphatic vessels opacification (arrow). (**B**) Subsequent opacification of the lymphatic network and deep lymph nodes (arrow). (**C**) After 81 min, opacification of the cisterna chyle occurred (arrow). (**D**) Deployment of 3 embolization coils (white arrow) in the thoracic duct proximal to the estimated site of leak (arrowhead—surgical clips from previous operations).

Conventional lymphangiography is an invasive imaging modality that was historically used to evaluate for lymphatic vessels and lymph node metastasis. Two techniques have been described for conventional lymphangiography: pedal and intranodal lymphangiography. Pedal lymphangiography starts by intradermal injection of methylene blue in the subcutaneous soft tissue. The dye is used to stain the lymphatic vessels. Afterward, a peripheral lymphatic vessel is cannulated with a very small needle and injected with a radiopaque oil contrast (Lipiodol) [48,49]. Intranodal lymphangiography is performed through directly injecting an inguinal lymph node with a radiopaque oil contrast (Lipiodol) followed by serial angiographic imaging of the area of concern [50,51]. Historically, CL has been utilized to evaluate for lymphatic vessels and lymph node metastasis. Normal lymph node homogeneously opacifies with the oil contrast agent. Presence of filling defects within the lymph node would be interpreted as a potential metastasis [52,53,54]. Currently, lymphangiography has been replaced by CT and MRI for cancer staging [9]. However, lymphangiography with or without thoracic duct embolization is still performed for diagnostic and therapeutic purposes in patients with postsurgical or posttraumatic lymphatic leaks, chylus ascites, or chylus pleural effusions (Figure 15 and Figure 16) [48,49,50].

#### 4.2.2. Magnetic Resonance Lymphangiogram (MRL)

MRL is a novel imaging technique that is used mainly to evaluate the lymphatic vessels in patients with lymphedema, which is commonly encountered in postsurgical lymph node dissection. Similar to conventional lymphangiography, either nodal or pedal approach is utilized for MRL [18,55,56]. In the pedal approach, a diluted gadolinium-based contrast media (GBCM) is injected intradermally in the interdigital web spaces or dorsal aspect of the foot [55,56]. In the nodal approach, the inguinal lymph nodes are directly injected with GBCM under US guidance [18] The following sequences are obtained: regular T2 weighted, heavily T2 weighted, precontract T1, and postcontrast T1 (THRIVE, VIBE, LAVA). Delayed imaging at 15–30 min after contrast injection can be performed if examination of the washout phase is required [18,55,56]. MRL without contrast has been reported in the literature. It is a heavily T2 weighted MR sequence similar to MR cholangiopancreatography and urography [57,58]. The main advantage of MRL over CL is absence of patients’ exposure to ionizing radiation. On the other hand, venous contamination and long scanning time are the technical challenges with MRL. Venous and lymphatic vessels can be differentiated on the basis of morphology. Lymphatic vessels are more tortuous and have a beaded appearance [18,56]. The main clinical application of MRL is for lymphedema evaluation. Abnormal lymphatic vessels are ectatic with multiple collaterals and retrograde flow of contrast in addition to subcutaneous soft tissue thickening due lymphedema. Contrast leakage in the peritoneum, pericardium, or pleural cavity can be identified [18,56].

#### 4.2.3. Ultrasmall Superparamagnetic Particles of Iron Oxide (USPIO) MRL

Despite being the cornerstone for cancer staging, conventional CT and MRI are limited in differentiating between benign and malignant lymph nodes, especially when lymph nodes are not enlarged. USPIO MRL is a novel imaging modality used to evaluate the lymphatic system. In cancer patients, USPIO MRL is performed using ultrasmall superparamagnetic iron oxide as the contrast agent. After intravenous injection, USPIO is filtered in the interstitium. Afterwards, USPIO is phagocytosed by macrophages and transported to reticuloendotilial organs including lymph nodes [59,60]. Twenty to thirty-six hours after intravenous injection of USPIO, heavily T2-weighted MR or gradient-echo T2*-weighted sequences are obtained. USPIO is a paramagnetic agent that changes the homogeneity of the magnetic field and produces signal loss, especially on susceptibility-sensitive sequences such as the gradient echo sequence. Normal lymph nodes will uptake USPIO and demonstrate low signal intensity on post-injection imaging. Malignant lymph nodes are infiltrated by malignant cells and lack normal lymphoid tissue and macrophages that normally uptake USPIO. Thus, malignant lymph nodes appear essentially unchanged after USPIO injection due to lack of USPIO uptake within the malignant lymph nodes [59,61,62]. The overall sensitivity, specificity, and accuracy of USPIO MRL are 90.5–100%, 37.5–97.8%, and 97.3%, respectively. However, USPIO MRL sensitivity, specificity, and accuracy for lymph node less than 5 mm are 41%, 98%, and 90% [60,63,64]. Despite the high specificity and sensitivity of USPIO MRL, the clinical adoption of this imaging modality worldwide is still in the very early phases due to advanced technologies required for developing USPIO. Additional challenges for USPIO include iron toxicity and long circulation time that requires a significant delay between contrast injection and image acquisition for up to 36 h.

### 4.3. Sentinel Lymph Node (SNL) Imaging

Accurate locoregional staging of various tumors significantly impacts patient management. Historically, draining lymph node dissection (LND) was the standard diagnostic procedure and treatment of choice for evaluating and treating locoregional lymph node metastasis. However, LND is invasive and associated with higher risk of lymphedema. With the introduction of sentinel lymph node imaging in the late 19th century, the rate of radical lymphatic dissection in melanoma patients has drastically declined. SNL imaging relies on the fact that all primary tumors are drained by lymphatics to a certain lymph node or a group of lymph nodes. Identification of SNL and surgical resection for pathologic evaluation can save patients unnecessary LND. Blue V dye was initially used to detect SNL in melanoma patients. The dye is injected intradermally and is transported by the lymphatic system. The draining lymphatic vessels and sentinel lymph nodes are visually identified by the blue color and resected for pathologic evaluation. On the basis of pathological findings, the decision to proceed with LND is made. Blue dye sentinel lymph node imaging is 96% accurate in detecting metastatic lymph nodes in melanoma patients [65,66]. The same technique has been applied in breast cancer, with a reported sensitivity of 88% [67].

#### Lymphoscintigraphy

**Figure 17:**
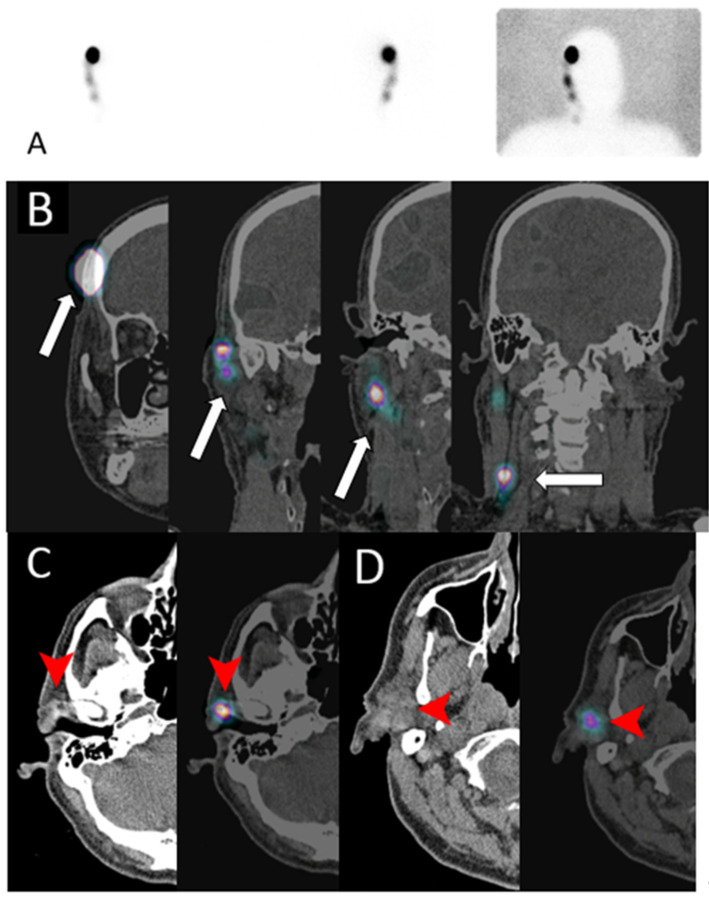
A 68-year-old male with melanoma in the right temporal region who underwent lymphoscintigraphy (**A**) and complementary SPECT/CT scan in the coronal plane (**B**); (**C**,**D**) show CT (left) and fused SPECT/CT images (right) for sentinel lymph nodes mapping. Intradermal injection of the TC^99m^ sulfur colloid in the right temple (arrow in (**B**)) shows tracer uptake in the pre-auricular region (arrowhead in (**C**)) and intra-parotid region (arrowhead in (**D**)), consistent with sentinel lymph drainage.

**Figure 18:**
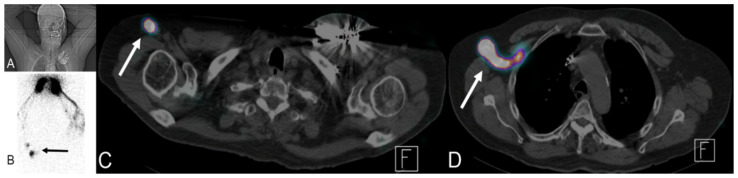
A 76-year-old male patient with a history of tongue base cancer status post-chemoradiation and left upper extremity lympho-venous bypass due to swelling of his left arm. Lymphoscintigraphy was performed by injection of sulfur colloid (radiotracer) into both hands. (**A**) The patient position. (**B**) Right anterior scintigraphy position with accumulation of tracer in both hands and tracer uptake in the right arm (black arrow). (**C**,**D**) Fused SPECT/CT with transit of tracer via proximal arm LN (white arrow in (**C**)) to reach lymph nodes at right axilla (white arrow (**D**)) with absence of any tracer uptake in the whole left upper extremity, confirming normal lymphatic drainage of the right arm with lymphedema of the left arm.

After the introduction of blue V dye SNL imaging, multiple nuclear radioisotopes have been used for lymphoscintigraphy (nuclear SNL): ^99m^Tc sulfur colloid, ^99m^Tc tilmanocept, ^99m^Tc nanocolloid of albumin, and ^99m^Tc antimony sulfide colloid. Performance of lymphoscintigraphy is dependent on physical characteristics of the radioisotope used. The preferred isotope should not diffuse in the interstitium or immediately transit into the venous system. The radiotracer should also move at an acceptable speed throughout the lymphatic vessels. Radioisotope particle size of 30 to 100 nm has been found to be optimal to achieve these requirements [68]. After intradermal injection, the radioisotope is transferred from the interstitium by the lymphatic capillaries into the lymphatic system and eventually to a draining lymph node. Sentinel lymph node is defined as the first draining lymph node receiving lymphatic drainage or afferent lymphatics from the primary tumor (Figure 17 and Figure 18) [69,70]. The scintigraphic appearance time (SAT), lymphatic transit rate (LTR), and scintigraphic saturation time (SST) are lymphoscintigraphic parameters that have been used to predict the probability of SLN metastasis in melanoma and breast cancer patients [71,72,73,74]. SAT is the time interval between radioisotope injection and visualization of the SLN. Short SAT has been associated with a higher probability of SNL metastasis, while long SAT carries lower likelihood of metastatic SLN. However, exact cutoff parameters defining long and short SAT vary. Short SAT has been reported to be less than 20 and 32 min [71,72,74]. SST represents the time needed for SNL to reach peak uptake. LTR is calculated by dividing the tumor to SNL distance over the SST. Patients with metastatic SNL have high LTR [73]. The correlation between high lymphatic flow and SNL metastasis can be explained by increased tumoral and peritumoral lymphangiogenesis, resulting in increased lymphatic flow [71,72,73]. In addition to melanoma and breast cancer, SNL lymphoscintigraphy is used and investigated in surgical planning applications for multiple malignancies including head and neck, valvular, cervical, and prostate cancers [75,76,77,78,79,80]. Utilizing SPECT/CT with lymphoscintigraphy improves anatomical localization and detection rate of lymphoscintigraphy [81,82]. Multiple factors can affect lymphoscintigraphy including BMI, age, and tumor location. Non-visualization rate of sentinel lymph node has been reported to be 2–28% [83].

## 5. Conclusions

Understanding the lymphatic system anatomy and physiology is crucial for evaluation of nodal and lymphatic metastasis. Multiple endothelial growth factors are involved in tumoral and peritumoral lymphangiogenesis that can be a potential target for cancer metastasis treatment. Multiple imaging modalities are available to evaluate lymphatic metastasis. Each imaging modality has its own potentials and limitations. Understanding potentials and limitations of these modalities is critical for optimal patient care.

## Figures and Tables

**Table 1:** US and Doppler features of benign and malignant lymph nodes. There is no sole criterion to define malignant lymph nodes. Constellation of multiple features suggest malignancy. Additionally, significant overlap between reactive and malignant lymphadenopathy remains.

US Criteria	Benign Lymph Node	Malignant Lymph Node
Size	<1 cm in short axis	≥1 cm in short axis
Shape	Oval or elliptical	Round
Border	Indistinct	Sharp
Echogenicity	Hypoechoic	Very hypoechoic
Hilum	Maintained fatty hilum	Absent fatty hilum
Vascularity	Avascular or hilar vascularity	Peripheral or mixed
Resistive index	Low	High
